# 5-Phenyl-10,15,20-Tris(4-sulfonatophenyl)porphyrin: Synthesis, Catalysis, and Structural Studies

**DOI:** 10.3390/molecules23123363

**Published:** 2018-12-19

**Authors:** Aitor Arlegui, Zoubir El-Hachemi, Joaquim Crusats, Albert Moyano

**Affiliations:** 1 Section of Organic Chemistry, Department of Inorganic and Organic Chemistry, University of Barcelona, Faculty of Chemistry, Carrer de Martí i Franquès 1-11, 08028 Barcelona, Catalonia, Spain; aitorarlegui@hotmail.com (A.A.); zelhachemi@ub.edu (Z.E.-H.); 2 Institute of Cosmos Science, Carrer de Martí i Franquès 1-11, 08028 Barcelona, Catalonia, Spain

**Keywords:** azotization, heterogeneous catalysis, cryo-TEM, deamination, Diels-Alder reaction, PFM imaging, porphyrin aggregates, porphyrin synthesis, reduction, sulfonation, water-soluble porphyrin

## Abstract

A convenient protocol for the preparation of 5-phenyl-10,15,20-tris(4-sulfonatophenyl)porphyrin, a water-soluble porphyrin with unique aggregation properties, is described. The procedure relies on the one-pot reductive deamination of 5-(4-aminophenyl)-10,15,20-tris(4-sulfonatophenyl)porphyrin, that can be in turn easily obtained from 5,10,15,20-tetraphenylporphyrin by a known three-step sequence involving mononitration, nitro to amine reduction and sulfonation of the phenyl groups. This method provides the title porphyrin in gram scale, and compares very favorably with the up to now only described procedure based on the partial sulfonation of TPP, that involves a long and tedious chromatographic enrichment of the final compound. This has allowed us to study for the first time both the use of its zwitterionic aggregate as a supramolecular catalyst of the aqueous Diels–Alder reaction, and the morphology of the aggregates obtained under optimized experimental conditions by atomic force microscopy and also by transmission electron cryomicroscopy.

## 1. Introduction

Water-soluble, sulfonated porphyrins and their metallated derivatives [1] have been extensively investigated due to their broad range of applicability, covering medicinal, analytical, and supramolecular chemistry. The trisodium salt of 5-phenyl-10,15,20-tris(4-sulfonatophenyl)porphyrin (TPPS_3_, **1**) has been the object of several biological [2,3], photophysical [4,5,6], and spectroscopic [7] studies in the past 50 years. On the other hand, the zwitterionic, diprotonated forms of sulfonated porphyrins give rise to supramolecular aggregates in aqueous solution, providing suitable models for natural light-harvesting complexes in photosynthesis [8,9]. Also, in this context, compound **1** presents an especial interest due to the distinct supramolecular behavior of its diprotonated form, compared to that of the readily available tetrasulfonated counterpart (TPPS_4_) [10,11,12,13,14,15]. In particular, it is worth noting that upon concentration of dilute solutions of porphyrin **1** in acidic aqueous media, non-racemic chiral supramolecular structures are obtained following a process which undergoes spontaneous mirror symmetry breaking; interestingly, the chiral outcome of this self-assembly process can be selected at will by the macroscopic chirality of the shear forces exerted by stirring. [10,11]

Currently, however, further studies using the sulfonated porphyrin **1** are often hampered by the amount of sample required to perform the envisaged experiments. Up to now, the sole synthetic method available for the preparation of **1** is based on the direct sulfonation of 5,10,15,20-tetraphenylporhyrin (TPP) with concentrated sulfuric acid [16,17,18,19]. Avoiding the harsh experimental conditions that lead to the exclusive formation of the tetrasulfonated product (100 °C, 6 h; rt, 12 h), milder sulfonation conditions (e.g., 4 °C, 24 h) afford mixtures of porphyrins differing both in the number of sulfonate groups and in their relative positions within the molecule. The relative amount of the trisulfonated compound can be maximized under carefully controlled reaction temperatures and times, as assessed by reverse-phase HPLC analysis of the sulfonation product [19]. Then, in the work-up procedure, most of the sulfuric acid can be conveniently removed by pouring the reaction crude onto a small amount of water and the mixture of aggregated sulfonated porphyrins is separated by centrifugation. Following neutralization, however, only after long and tedious purification by reverse-phase column chromatography (C18) using methanol-phosphate buffer mixtures as eluent, followed by iterative enrichment of the desired chromatographic fractions, small amounts of **1** (typically 10–20 mg starting from 1 g of TPP) can be isolated in pure form after desalting. It is also worth noting that an alternative procedure based on Lindsey’s method [20] for the mixed condensation of 4-trimethylsilylbenzaldehyde and benzaldehyde with pyrrole followed by desilylative sulfonation [21], that has been used by Tkachenko, Imamori et al. for the preparation of 5-(4-alkoxyphenyl)-10,15,20-tris(4-sulfonatophenyl)porphyrins (in 70 mg scale) [22], would be utterly impractical for the synthesis of **1** owing to the very difficult separation of the mixture of 4-silylated derivatives of TPP obtained in the first step.

In the course of our current interest on the chemistry of sulfonated porphyrins [23], we were in need of substantial amounts of porphyrin **1** and we decided to develop a more direct approach to this compound, avoiding the above-mentioned drawbacks would allow for the first time its preparation at the gram scale.

## 2. Results and Discussion

### 2.1. Reductive Deamination Route for the Gram-Scale Synthesis of Porphyrin ***1***

**Scheme 1:**
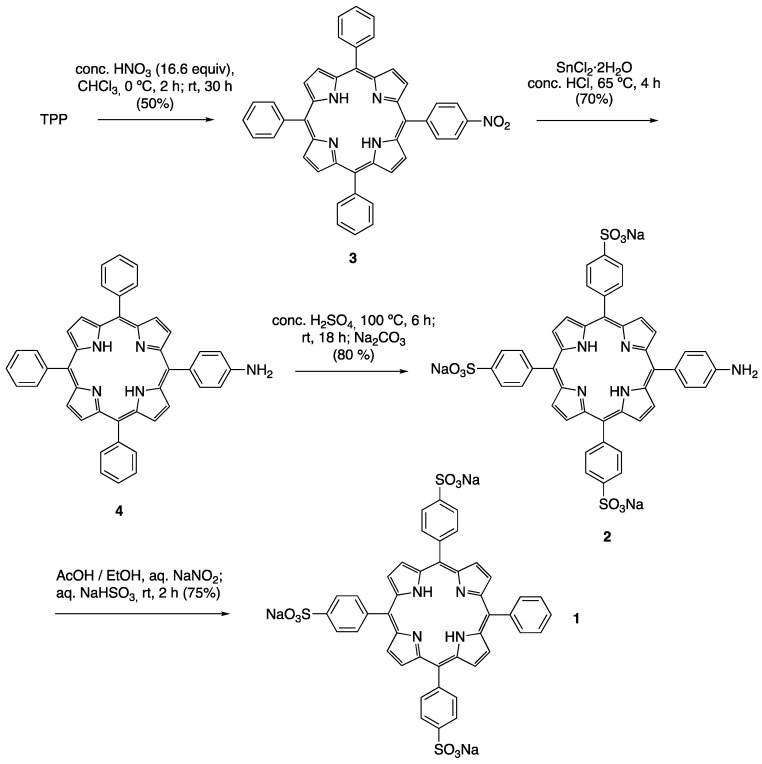
Synthesis of 5-phenyl-10,15,20-tris(4-sulfonatophenyl)porphyrin sodium salt (**1**).

We easily identified that the reductive deamination of the known 5-(4-aminophenyl)-10,15,20-tris(4-sulfonatophenyl)porphyrin **2** [24], if successful, would provide a straightforward route for the synthesis of 5-phenyl-10,15,20-tris(4-sulfonatophenyl)porphyrin **1**. It is worth noting, however, that the typical two-step procedure for the removal of the amine group in anilines, involving diazotization followed by reduction by hypophosphorous acid [25], cannot be directly applied to the sulfonated porphyrin **2**, that gives rise to a highly insoluble (at preparative concentrations) aggregate in the strongly acidic aqueous medium required for the formation of the diazonium salt. We decided however to investigate this route, hoping that—as it turned out to be the case—a way to circumvent this difficulty would be possible. Our synthetic procedure is summarized in Scheme 1.

The preparation of the sulfonated aminoporphyrin **2** took place uneventfully, following closely the procedure described by Kruper et al. [24]. Mononitration of TPP was best affected by the dropwise addition of a controlled amount (16.6 equiv.) of fuming nitric acid at 0–5 °C during a 2 h period, followed by stirring the reaction mixture at rt until the disappearance of the starting material. After chromatographic purification, the desired 5-(4-nitrophenyl)-10,15,20-triphenyl porphyrin **3** was routinely obtained in 1 g batches in a 50% yield. Tin(II) chloride-mediated reduction and subsequent sulfonation of the phenyl groups in **4** gave access to the key porphyrin **2**. We were then faced with the critical reductive deamination step of this compound, but after a literature survey of previously described procedures and some experimentation, we were delighted to find that the one-pot method of Meier et al. [26] was perfectly suited to our purpose, affording **1** in 0.70 g batches with 80% yield. Most probably, the success of this procedure is due to the fact that the diazotization and reduction steps are performed in an EtOH/AcOH solvent mixture, in which the formation of aggregates does not take place in significant amounts.

**Figure 1:**
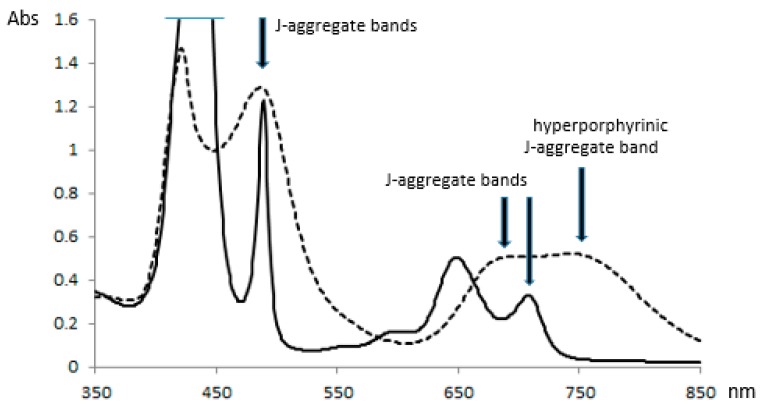
UV-vis spectra of **1** (continuous line; c = 2.0 × 10^−4^ M) and of its synthetic precursor **2** (dashed line; c = 1.8 × 10^−5^ M) in water at pH = 4.0 (acetic acid/sodium acetate buffer 0.1 M), cell path = 1 cm.

The conversion of **2** into TPPS_3_ (**1**) can be easily assessed by UV-vis spectroscopy (Figure 1). At pH = 4.0, **2** has both its pyrroleninic nitrogen atoms protonated while the peripheral amino group is still in its basic form, which leads to the formation of aggregated species showing a distinct far-red shifted hyperporphyrin band in its aggregated species [27], that is not present in those of the deaminated porphyrin **1**.

**Figure 2:**
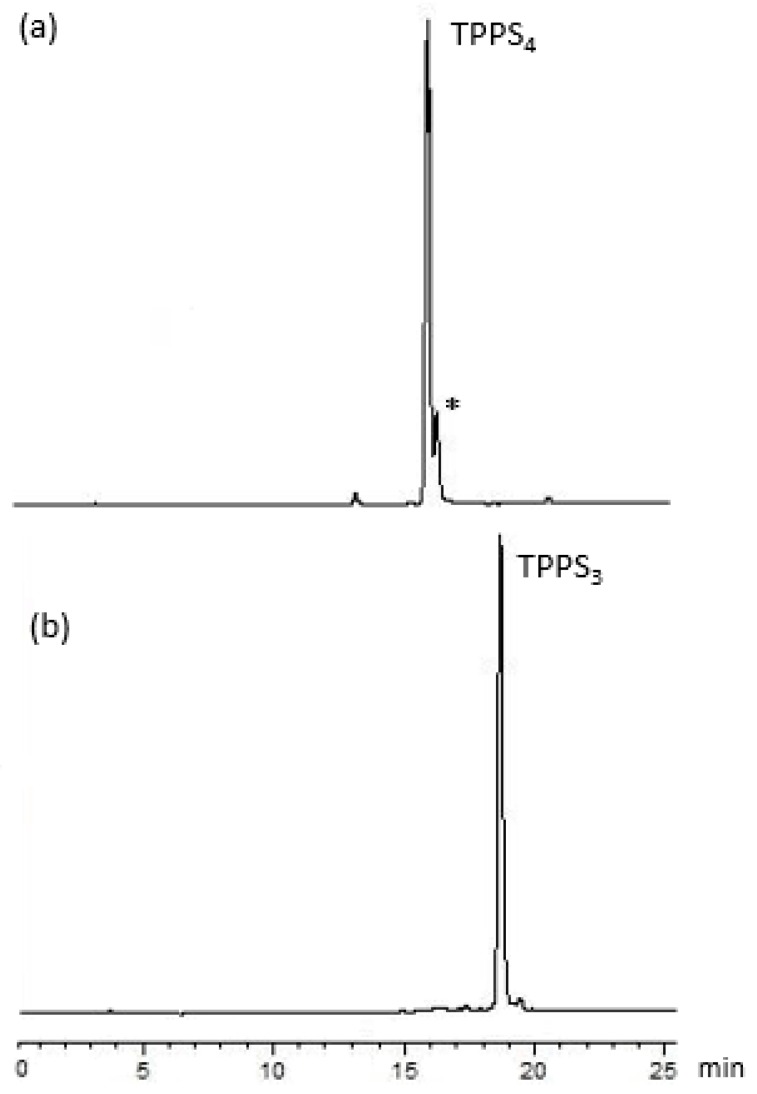
Reverse-phase HPLC analysis of TPPS_4_ (**a**) and TPPS_3_ (**1**) (**b**) samples. The impurity (*) in (**a**) corresponds to the regioisomer with one of the sulfonate groups in the meta position; the analogous impurity in the trisulfonated counterpart can be more easily separated in the desalting process to afford pure fractions of **1**. See the Materials and Methods Section for the detailed chromatographic conditions and procedures.

As depicted in Figure 2, in which we compare the reverse-phase HPLC chromatograms of 5,10,15,20-tetrasulfonatophenylporphyrin (**a**, TPPS_4_) with that of **1** (TPPS_3_, **b**), the product is obtained in essentially pure form, since the small quantities of meta-sulfonated regioisomers [28] formed in the sulfonation step can easily be separated in the desalting process.

Finally, when required for structural studies of the aggregated species, the cation-free diprotonated form of **1** ([TPPS_3_^−^][H_3_O^+^]) could be obtained at the 0.2 g scale by treatment of the trisodium salt (TPPS_3_Na_3_) with 0.1 M aq. HCl, centrifugation of the resulting suspension at 6000 rpm, and washing of the precipitate with Milli-Q^®^ (Merck Millipore, Billerica, MA, USA) water until a constant pH of 0.85, followed by lyophilization. Note that the porphyrin in this acidic form regulates by itself the pH of the medium and thus its inner-core acid–base equilibria, and hence its aggregation state, are dictated by its own concentration.

### 2.2. Heterogeneous Supramolecular Catalysis of the Aqueous Diels–Alder Reaction by TPPS_3_ (***1***) Aggregates 

**Figure 3:**
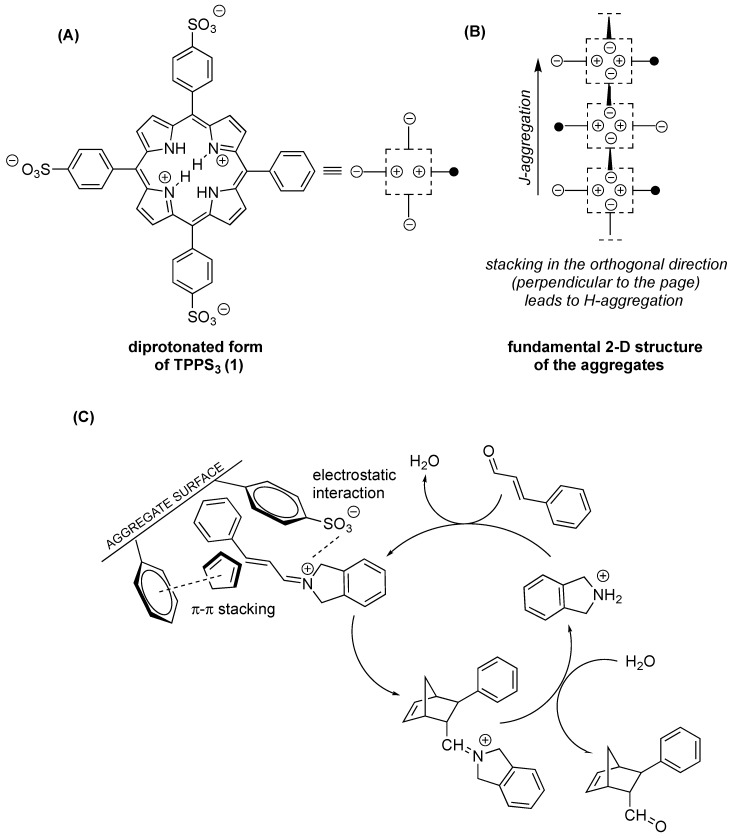
(**A**,**B**) Fundamental structure of the *J*-aggregates of diprotonated TPPS_3_ (**1**); (**C**) Catalysis of the Diels-Alder reaction by the heteroaggregates of general formula ([TPPS_3_^−^][R_2_NH_2_^+^])_x_.

Whereas metallated porphyrins have been extensively exploited both in homogeneous [29] and in heterogenous catalysis [30,31], the catalytic properties of non-metallated porphyrins remain up to date virtually unexplored [32]. The fundamental structure of the aggregates of diprotonated TPPS_3_ (1) [12] correspond to a two-dimensional (2D) assembly (*J*- and *H*-aggregates) of zwitterions stabilized by electrostatic bonding (between the dicationic core of one porphyrin and two sulfonate anions of the adjacent monomers) and hydrophobic interactions (Figure 3A,B), similarly as in the case of TPPS_4_ [33]. These supramolecular assemblies can be regarded as a noncovalent polymeric sulfonic acid, requiring a countercation (sodium or hydronium cation) per each monoanionic monomer to achieve electroneutrality. We hypothesized that, taking advantage of the supramolecular structure, if these cations could be replaced by α,β-unsaturated iminium ions derived from cinnamaldehyde and a cyclic secondary amine, interactions between the phenyl groups of the porphyrin and a conjugated diene such as cyclopentadiene could bring these two species into near proximity at the aggregate surface, so that the *J*-aggregates of TPPS_3_ (**1**) would act as heterogeneous organocatalysts of ‘on water’ Diels–Alder reactions. (Figure 3C). 

**Scheme 2:**
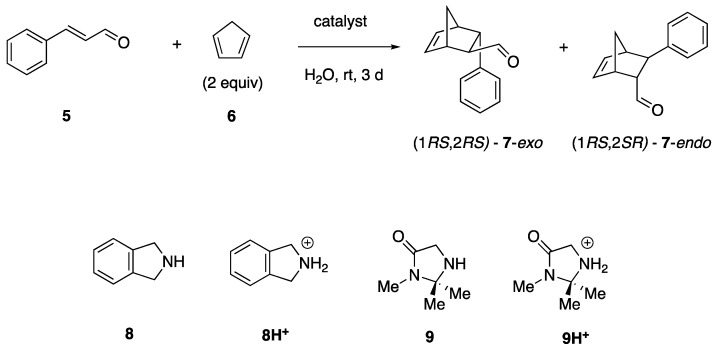
Aqueous Diels-Alder reaction between cinnamaldehyde (**5**) and cyclopentadiene (**6**) catalyzed by salts of cyclic secondary amines **8** and **9**.

We proceeded therefore to test the catalysis of the aqueous Diels-Alder reaction between cinnamaldehyde (**5**) and cyclopentadiene (**6**) [34] by the zwitterionic heteroaggregates derived from TPPS_3_ (**1**) and cyclic secondary amines (Scheme 2 and Table 1).

In the first place, taking into account the acceleration of Diels-Alder reactions in the presence of water [35], we performed the reaction in the absence of any catalyst, and we found a maximum 2% yield of the adducts **7** after three days at rt (entry 1 in Table 1). We examined next the catalytic efficiency of the *p*-toluenesulfonate salts of the same amines (**8** and **9**) that we would use in conjunction with the aggregates of the diprotonated porphyrin [TSPP_3_^−^][H_3_O^+^] (entries 2 and 3, respectively). We recorded yields higher than 50%, confirming the viability of homogeneous organocatalysis of the aqueous Diels-Alder reaction by sulfonate salts of either isoindolinium (**8H^+^**) or imidazolidinonium (**9H^+^**) cations. Finally, we prepared the desired heteroaggregates of general formula [TPPS_3_^−^][R_2_NH_2_^+^] in the following way: A 0.0375 M aqueous solution of trisodium salt of 1 (TPPS_3_ Na_3_) was treated with 1.5 molar equivalents of sulfuric acid, giving rise to a suspension of the aggregates of the diprotonated species [TPPS_3_^−^][H_3_O^+^]. Upon careful addition of one molar equivalent of either isoindoline (**8**) or of 2,2,3-trimethylimidazolidinone (**9**), we observed no dissolution of the dark green precipitate, indicating the formation of the heteroaggregates of [TPPS_3_^−^][R_2_NH_2_^+^] while keeping the porphyrin in its deprotonated form. We were pleased to find that these heterogeneous mixtures also showed catalytic activity in the Diels-Alder reaction (19% yield for isoindoline **8**, entry **4**; 7% yield for imidazolidinone **9**, entry 5). Especially in the case of isoindoline **8**, the *exo*:*endo* ratio is clearly different from that observed in solution (compare entries 2 and 4), which supports the case of heterogeneous catalysis under these conditions. To the best of our knowledge, this is the first example of the use of a non-metallated porphyrin supramolecular aggregate as a catalyst of a chemical reaction. Moreover, after extraction of the Diels-Alder adducts **7** and of unreacted starting materials with an organic solvent, the porphyrin 1 can be easily purified and recovered from the aqueous phase by centrifugation, neutralization with Na_2_CO_3_, reverse phase column chromatography and lyophilization.

It is worth noting here that these reactions were run at a 0.075 mmol scale of the catalyst, requiring *circa* 70 mg of TPPS_3_Na_3_ for each run. Clearly, this study has been only possible thanks to the gram-scale synthesis of **1** described in the previous section.

### 2.3. Atomic Force and Transmission Electron Microscopy Study of the Morphology of TPPS_3_ (***1***) Aggregates

**Figure 4:**
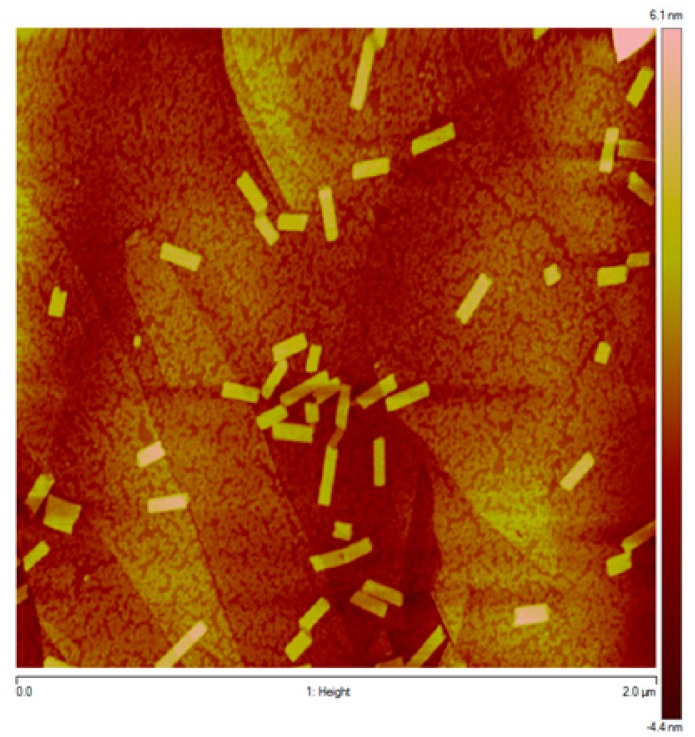
Peak force microscopy imaging of nanotubes of TPPS_3_ (**1**) aggregates deposited on HOPG.

**Figure 5:**
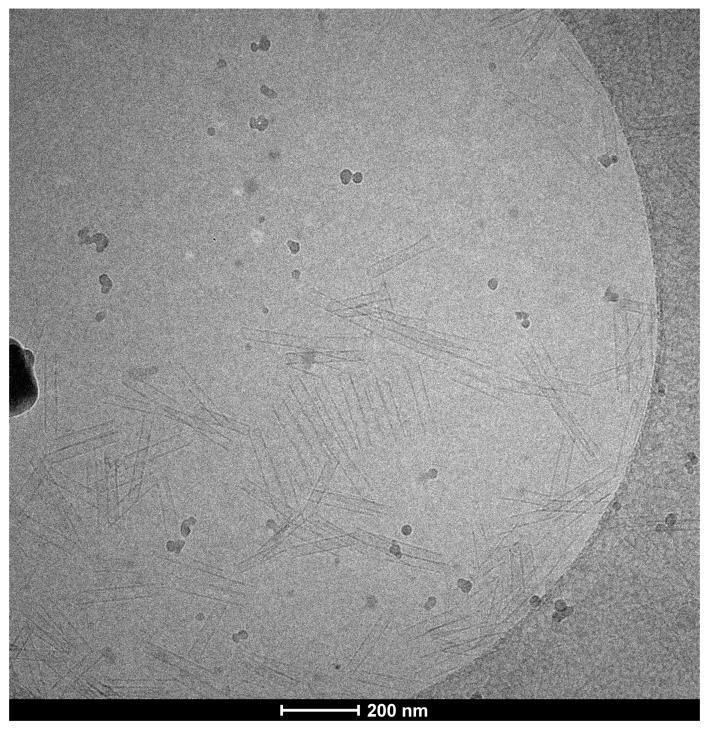
Cryo-TEM image of TPPS_3_ (**1**) aggregates (the black drops correspond to the initial phase of the transformation of vitrified water to crystallized water).

The morphology of the aggregated species obtained by carefully controlled capillary injection of concentrated mother solutions of the cation-free zwitterionic porphyrin 1 (see Materials and Methods) was studied by peak forced microscopy (PFM) of samples deposited onto highly oriented pyrolytic graphite (HOPG) and by transmission electron cryomicroscopy (cryo-TEM). PFM imaging (Figure 4) showed collapsed straight particles (bilayered ribbon-like structures), which cryo-TEM revealed to be hollow nanotubes (Figure 5).

It is again important to observe that these morphological characterization studies have been greatly facilitated by the availability of porphyrin 1 in large amounts, since the morphologies of the aggregated species proved to be highly dependent on the exact experimental procedure used in their preparation, which required careful optimization and a large number of experiments.

In summary, we have developed a simple and convenient route to the highly valuable 5-phenyl-10,15,20-tris(4-sulfonatophenyl)porphyrin **1**, that has allowed for the first time its preparation in gram quantities and that has therefore paved the way for the further study of its remarkable properties both from the catalysis and from the structural points of view.

## 3. Materials and Methods 

### 3.1. General Methods

Commercially available reagents, catalysts, and solvents were used as received. Water of Millipore Q quality (18.2 MΩ·cm, obtained from Milli-Q1 Ultrapure Water Purification Systems, Merck Millipore, Billerica, MA, USA) was used. ^1^H (400 MHz) NMR spectra were recorded with a Varian Mercury 400 spectrometer. Chemical shifts (δ) are given in ppm relative to the solvent peak of tetramethylsilane (δ = 0.00 ppm), and coupling constants (*J*) are given in Hz. The spectra were recorded at room temperature. TMS served as an internal standard. Data are reported as follows: s, singlet; d, doublet; t, triplet; q, quartet; m, multiplet; br, broad signal. IR spectra were obtained in a Nicolet 6700 FTIR instrument (Thermo Fisher Scientific, Walham, MS, USA), using ATR techniques. UV-vis spectra were recorded on a double-beam Cary 500-scan spectrophotometer (Varian Medical Systems, Palo Alto, CA, USA); cuvettes (quartz QS Suprasil, Hellma Analytics, Müllheim, Germany) cm were used for measuring the absorption spectra. pH measurements were performed on a CRISON Micro pH 2000 pH-meter (Crison 52-04 glass electrode) at room temperature. The pH-meter was calibrated prior to each measurement with buffers at pH = 7.00 and 4.00 (Metrohm AG, Herisau, Switzerland). Thin-layer chromatography was carried out on silica gel plates Merck 60 F_254_, and compounds were visualized by irradiation with UV light. Flash column chromatography was performed using silica gel Merck 60 (particle size: 0.040–0.063 mm, Merck Millipore, Billerica, MA, USA).

The HPLC analyses were performed on a Shimadzu high-performance liquid chromatograph equipped with two LC-10AS pumps, a Shimadzu CBM controller, an analytical precolumn Resolve C18 (Waters Corporation, Milford, MA, USA), and a Nucleosil 120-5C18 analytical column, using an elution gradient consisting of a mixture of methanol and tetrabutylammonium phosphate buffer (3 mmol·L^−1^; pH = 7.0) (1:1 *v*:*v*) to pure methanol over a period of 30 min at a flow rate of 0.6 mL min^−1^ (~2700–1050 psi). All solvents were of HPLC grade and were carefully degassed prior to use. At time 0 the sample was injected. The elution profile was monitored at λ = 414 nm (UV-vis detector SPD-6AV).

Peak force microscopy (PFM) images were acquired on a multimode atomic force microscope attached to a Bruker^®^ Nanoscope V electronics unit (Bruker Corporation, Billerica, MA, USA). The imaging mode used for these experiments was scan-assist tapping mode: in this operating mode, the scanning probe performed individual force curves on the sample surface, while maintaining a constant vertical force. The vertical force was set at 10–20 pN, which was a suitable threshold to ensure that sample deformation due to contact of the scanning probe with the sample was kept to a minimum. The scanning probe used in this study was a Bruker^®^ SLN-10 triangular probe made of silicon nitride, the spring constant of which was 0.35 N·nm^−1^. For the solution deposition procedure for PFM imaging on dry substrate see more details in reference [12].

Cryo-TEM images of the J-aggregates of TPPS_3_ (**1**) in aqueous solution were obtained as follows: A thin aqueous film was formed by dipping and withdrawing a bare specimen grid from the suspension. Glow-discharged holey carbon grids were used. After withdrawal from the suspension, the grid was blotted against filter paper, leaving thin sample films spanning the grid holes. These films were vitrified by plunging the grid into ethane, which was kept at its melting point by liquid nitrogen, by using a Vitrobot^®^ (FEI Company, Eindhoven, North Braban, Netherlands), and keeping the sample before freezing at 100% humidity. Vitrification of the thin films was initiated at room temperature. The vitreous sample films were transferred to a Tecnai F20 microscope (FEI Company, Eindhoven, North Braban, NL) by using a Gatan cryotransfer. The images were taken at 200 kV between −170 and −175 °C and by using low-dose imaging conditions.

TPP was prepared by the standard Adler-Longo procedure in propionic acid [36] and recrystallized from CH_2_Cl_2_/MeOH. NMR spectra for compounds are in the Supplementary Materials.

### 3.2. Synthetic Procedures and Product Characterization

*5-(4-Nitrophenyl)-10,15,20-triphenylporphyrin***3** [24]. In a 500 mL round-bottomed flask, equipped with magnetic stirring, 5,10,15,20-tetraphenylporphyrin (2.00 g, 3.25 mmol) was dissolved in 300 mL of chloroform and stirred under nitrogen. Yellow fuming nitric acid (3.4 g, 54 mmol) was added dropwise to the stirred solution of porphyrin at 0–5 °C through a pressure-equalizing dropping funnel over a 2 h period, and the resulting mixture was stirred at rt until total conversion of the starting material (30 h, TLC monitoring). The dark green solution was washed with water (5 × 100 mL) and was dried over anhydrous sodium sulfate. After filtration, the solution was concentrated under reduced pressure to 75 mL and was submitted directly to chromatographic purification (silica gel column), eluting with hexane/CH_2_Cl_2_ (1/1), to afford the mononitrated porphyrin **3** as a purple-colored solid (1.07 g, 50% yield). Spectral data coincided with those reported in the literature [24].^1^H-NMR (400 MHz, CDCl_3_) δ: 8.87 (d, 2H, *J* = 5.0 Hz, β-pyrrole), 8.86 (s, 4H, β-pyrrole), 8.74 (d, 2H, *J* = 5.0 Hz, β-pyrrole), 8.63 (d, 2H, *J* = 8.9 Hz, nitrophenyl), 8.40 (d, 2H, *J* = 8.9 Hz, nitrophenyl), 8.23–8.20 (m, 6H, *o*-phenyl), 7.82–7.73 (m, 9H, m-/p-phenyl), −2.77 (s, 2H, pyrrole NH).

*5-(4-Aminophenyl)-10,15,20-triphenylporphyrin***4** [24]. In a 50 mL round-bottomed flask, equipped with magnetic stirring, nitrophenyl porphyrin 3 (0.75 g, 1.14 mmol) was dissolved in concentrated aqueous HCl (25 mL) and stirred under nitrogen. Tin (II) chloride dihydrate (0.77 g, 3.42 mmol) was added to the solution in one portion, and the reaction was heated to 65 °C for 4 h. The porphyrin solution was then cooled at rt and poured over ice-water (35 mL), and the pH was adjusted to 8 with concentrated ammonium hydroxide. The aqueous solution was extracted with chloroform (6 × 20 mL), and the combined organic phases were dried over anhydrous sodium sulfate. After filtration, the solution was concentrated under reduced pressure to 20 mL and was submitted directly to chromatographic purification (silica gel column), eluting with CH_2_Cl_2_, to afford the desired porphyrin **4** as a purple-colored solid (0.50 g, 70% yield). Spectral data coincided with those reported in the literature [24]. ^1^H-NMR(400 MHz, CDCl_3_) δ: 8.93 (d, 2H, *J* = 5.0 Hz, β-pyrrole), 8.84 (d, 2H, *J* = 5.0 Hz, β-pyrrole), 8.83 (s, 4H, β-pyrrole), 8.24–8.20 (m, 6H, *o*-phenyl), 8.00 (d, 2H, *J* = 8.2 Hz, *m*-4-aminophenyl), 7.79–7.72 (m, 9H, *m*-/*p*-phenyl), 7.06 (d, 2H, *J* = 8.2 Hz, *o*-4-aminophenyl), 4.04 (s, 2H, amino), −2.75 (s, 2H, pyrrole NH).

*5-(4-Aminophenyl)-10,15,20-tris(4-sulfonatophenyl)porphyrin trisodium salt*, **2** [24]. In a 100 mL round-bottomed flask, equipped with magnetic stirring and a reflux Dimroth condenser capped with a calcium chloride tube, aminophenyl porphyrin **4** (0.60 g, 0.95 mmol) was placed, and concentrated H_2_SO_4_ (25 mL) was slowly added. The reaction was heated to 100 °C during 6 h and then it was left at rt during 18 h. After the addition of H_2_O (25 mL) the resulting green suspension was centrifuged at 6000 rpm during 30 min. The supernatant was decanted and the remaining sulfuric acid was neutralized with solid Na_2_CO_3_ to afford a purple-colored solution. Inorganic salts were removed by medium pressure reverse phase column chromatography using MCI GEL CHP20P (Diaion^®^, Supelco/Sigma-Aldrich, Billerica, MA, USA), in which the porphyrin is slightly retained thus allowing the elimination of inorganic salts using water as the eluent; increasing gradients of methanol (from 0% to 50%) conveniently eluted the porphyrin. Evaporation of the solvents under reduced pressure followed by lyophilization afforded the porphyrin **2** (trisodium salt, 0.71 g, 80% yield) as a purple-colored solid. Spectral data coincided with those reported in the literature [24].^1^H-NMR(400 MHz, DMSO-*d*_6_) δ: 9.04 (d, 2H, *J* = 5.1 Hz, β-pyrrole), 8.89 (s, 4H, β-pyrrole), 8.88 (d, 2H, *J* = 5.1 Hz, β-pyrrole), 8.26 (d, 6H, *J* = 8.2 Hz, 4-sulphonatophenyl), 8.14 (d, 6H, *J* = 8.2 Hz, 4-sulphonatophenyl), 7.93 (d, 2H, *J* = 8.4 Hz, 4-aminophenyl), 7.09 (d, 2H, *J* = 8.4 Hz, 4-aminophenyl), 5.62 (s, 2H, amino NH_2_), −2.83 (s, 2H, pyrrole NH).

*5-Phenyl-10,15,20-tris(4-sulfonatophenyl)porphyrin trisodium salt*, **1**. In a 250 mL round-bottomed flask, equipped with magnetic stirring, porphyrin trisodium salt **2** (1.00 g, 1.09 mmol) was disposed, and absolute ethanol (44 mL) and acetic acid (6.5 mL, 6.8 g, 114 mmol) were added at rt. To the resulting suspension, a solution of NaNO_2_ (0.75 g, 10.9 mmol) in 13 mL of water (13 mL) was added dropwise, followed by the addition of a solution of NaHSO_3_ (1.10 g, 10.9 mmol) in water (17.5 mL). At this point, the reaction mixture changed from a suspension to a solution, with the usual red porphyrin color. The reaction was stirred at rt for 2 h, and the ethanol was evaporated under reduced pressure. An aqueous HCl 0.1 M solution (25 mL) was added and the resulting green suspension was centrifuged at 6000 rpm during 30 min. The supernatant was decanted and the procedure was repeated once again. The medium was neutralized with solid Na_2_CO_3_ to afford a purple-colored solution. Inorganic salts were removed by medium pressure reverse phase column chromatography using MCI GEL CHP20P (Supelco/Sigma-Aldrich, Billerica, MA, USA), in which the porphyrin is slightly retained thus allowing the elimination of inorganic salts using water as the eluent; increasing gradients of methanol (from 0% to 50%) conveniently eluted the porphyrin. Evaporation of the solvents under reduced pressure followed by lyophilization afforded the porphyrin 1 (trisodium salt, 0.75 g, 75% yield) as a purple solid. FT-IR (cm^−1^): 3396, 1645, 1634, 1175, 1121, 1035, 1010, 965, 795, 736, 629. ^1^H-NMR(400 MHz, DMSO-*d*_6_) δ: 8.90–8.80 (m, 8H, β-pyrrole), 8.30–8.27 (m, 2H, *o*-phenyl), 8.25–8.21 (part AA’ of AA’BB’ system, 6H, 4-sulfonatophenyl), 8.12-8.08 (part BB’ of AA’BB’ system, 6H, 4-sulfonatophenyl), 7.92–7.87 (m, 3H, *m*-/*p*-phenyl), –2.87 (s, 2H, pyrrole NH). ^13^C-NMR (100.6 MHz, DMSO-*d*_6_) δ: 148.13, 148.11, 141.8, 141.6, 134.7, 134.2, 133–131 (unresolved broad signal), 128.6, 127.5, 124.7, 120.6, 120.12, 120.09. MS-ESI (negative), *m*/*z*: 853.12 (z = 1); calculated for C_44_H_29_N_4_O_9_S_4_: 853.10. 426.05 (z = 2); calculated for (C_44_H_28_N_4_O_9_S_4_)/2: 426.05. 283.70 (z = 3); calculated for (C_44_H_27_N_4_O_9_S_4_)/3: 283.67. UV–vis (H_2_O, 3.7 × 10^−6^ M, λ_max_ nm, log(ε) cm^−1^·mol^−1^): 413 (5.69), 517 (4.23), 554 (3.97), 580 (3.88), 637 (3.67).

To get the porphyrin **1** in its zwitterionic, cation-free form, the trisodium salt (200 mg, 0.22 mmol) was dissolved in aqueous 0.1 M hydrochloric acid (15 mL), and was centrifuged at 6000 rpm during 30 min; then the supernatant was decanted and the precipitate was washed with Milli-Q^®^ water until a constant pH of 0.85 of the washings was achieved. Finally, the porphyrin was lyophilized to give 158 mg (85% yield) of a dark green-colored solid. Alternatively, small amounts of freshly prepared cation-free zwitterionic species of porphyrin **1** can be obtained from the trisodium salt using a cation exchange column with a DOWEX resin (H^+^-form).

### 3.3. Representative Procedure for the Catalysis of the Diels-Alder Reaction by the [zw-TPPS_3_·(amine)] Heteroaggreates

In a typical experiment, first an aqueous solution of the 1:1 zw-TPPS_3_: Amine heteroaggreate was prepared as follows. In a 10 mL round-bottomed flask, 2 mL of water were added over 69 mg (0.075 mmol) of Na_3_TPPS_3_, and concentrated H_2_SO_4_ (11 mg, 8.6 µL, 0.1125mmol) was subsequently added, resulting in a thick dark-green suspension of the porphyrin aggregate. Over this suspension, isoindoline **8** (9 mg, 8.5 μL, 0.075 mmol) was added under vigorous magnetic stirring and the mixture was left stirring for further 10 min. Then, freshly distilled (*E*)-cinnamaldehyde **5** (66 mg, 63 μL, 0.50 mmol) and freshly distilled cyclopentadiene **6** (99 mg, 126 μL, 1.50 mmol) were sequentially added to the aqueous suspension of the catalyst, and the resulting heterogenous mixture was left to react under vigorous magnetic stirring. After 72 h, the reaction crude was poured onto water (7 mL), and the mixture was extracted with dichloromethane (4 × 10 mL). The combined organic phases were thoroughly washed with a saturated NaHCO_3_ solution (2 × 15 mL). Finally, the solution was dried over Na_2_SO_4_ and the organic solvent was eliminated under reduced pressure affording the crude mixture of Diels-Alder adducts **7** (109 mg) as a dark-yellow oil. In order to determine the conversion of the reaction and the diastereomeric ratio of the products, this crude mixture was analysed by ^1^H-NMR.

### 3.4. Procedure for the Preparation of Solutions TPPS_3_ (**1**) Nanotubes for PFM and Cryo-TEM Imaging

The required samples were prepared by the injection of 500 µL of a mother solution of the concentrated porphyrin (≈ 6 mM in water, equilibrated for 24 h obtained from freshly prepared 1 in its cation-free zwitterionic form) with a capillary needle (Hamilton syringe^®^ 725 needle; external diameter of 0.7 mm; injection time 5 s) in the bulk of an aqueous 0.01 M HCl solution. The syringe was immediately cleaned using methanol, a solution of saturated sodium hydrogen carbonate, and distilled water. The syringe metal parts do not seem to be affected by the short contact times with the acidic solution. 

## Figures and Tables

**Table 1:** Catalysis of the aqueous Diels-Alder reaction by ammonium salts and by TPPS_3_: Amine heteroaggregates (Scheme 2) ^a^.

Entry	Catalyst (mol%)	Yield ^b^	7*-exo*:7-*endo* ^b^
1	none	<2%	52:48
2	**8H^+^** TsO^−^ (30 mol%)	75%	70:30
3	**9H^+^** TsO^−^ (30 mol%)	50%	57:43
4 ^c^	[**8H^+^** TPPS_3_^−^]_x_ (15 mol%)	19%	60:40
5 ^c^	[**9H^+^** TPPS_3_^−^]_x_ (15 mol%)	7%	56:44

^a^ The reaction was conducted by using (*E*)-cinnamaldehyde **5** (0.50 mmol), cyclopentadiene **6** (1.5 mmol), amine (0.075 mmol), TPPS_3_ Na_3_ (0.075 mmol) and conc. H_2_SO_4_ (0.113 mmol) under stirring at room temperature for three days in water (2 mL). ^b^ Yield and *exo*:*endo* diastereomer ratio were determined from the integration of the aldehyde proton signals in the 400 MHz ^1^H-NMR spectra of the reaction crudes. ^c^ The results correspond to the arithmetic mean of three independent experiments.

## Supplementary Materials

The following are available online, NMR spectra for compounds **1**, **2**, **3**, **4**, and **7a/b** (Diels-Alder reaction crude).
