# Supplementary material for: 5-Phenyl-10,15,20-Tris(4-sulfonatophenyl)porphyrin: Synthesis, Catalysis, and Structural Studies
Source: Molecules. 2018 Dec 19;23(12):3363. doi: 10.3390/molecules23123363 (PMC6321080; doi:10.3390/molecules23123363)

# 5-Phenyl-10,15,20-tris(4-sulfonatophenyl)porphyrin: synthesis, catalysis and structural studies

**Aitor Arlegui [1], Zoubir El-Hachemi [1,2], Joaquim Crusats [1,2,*] and Albert Moyano [1,*]**

1 Section of Organic Chemistry, Department of Inorganic and Organic Chemistry, University of Barcelona, Faculty of Chemistry, c. Martí i Franquès 1-11, 08028-Barcelona, Catalonia, Spain
2 Institute of Cosmos Science, c. Martí i Franquès 1-11, 08028-Barcelona, Catalonia, Spain
* Correspondence: j.crusats@ub.edu (J. Crusats); amoyano@ub.edu, Tel.: +34-93-4021245 (A. Moyano)

SUPPORTING INFORMATION

NMR spectra for compounds **1 2**, **3**, **4**, and **7a/b** (Diels–Alder reaction crude)

*5-(4-Nitrophenyl)-10,15,20-triphenylporphyrin* **3**

$^{1}$H NMR (400 MHz)

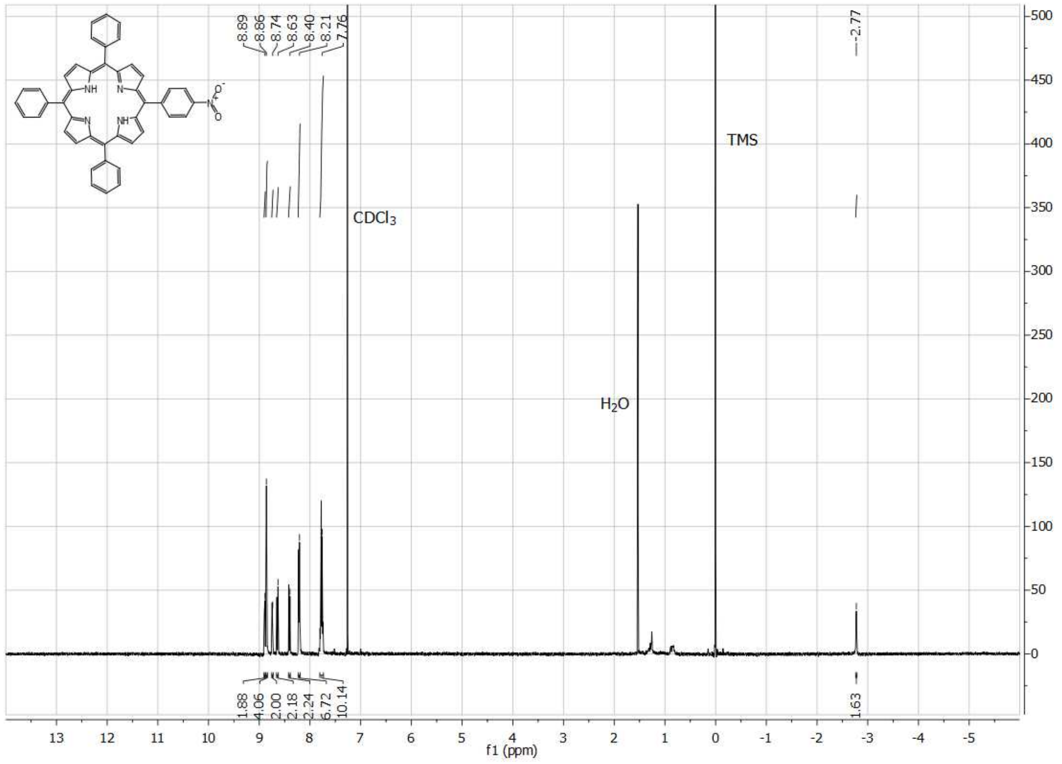

*5-(4-Aminophenyl)-10,15,20-triphenylporphyrin* **4**

$^{1}$H NMR (400 MHz)

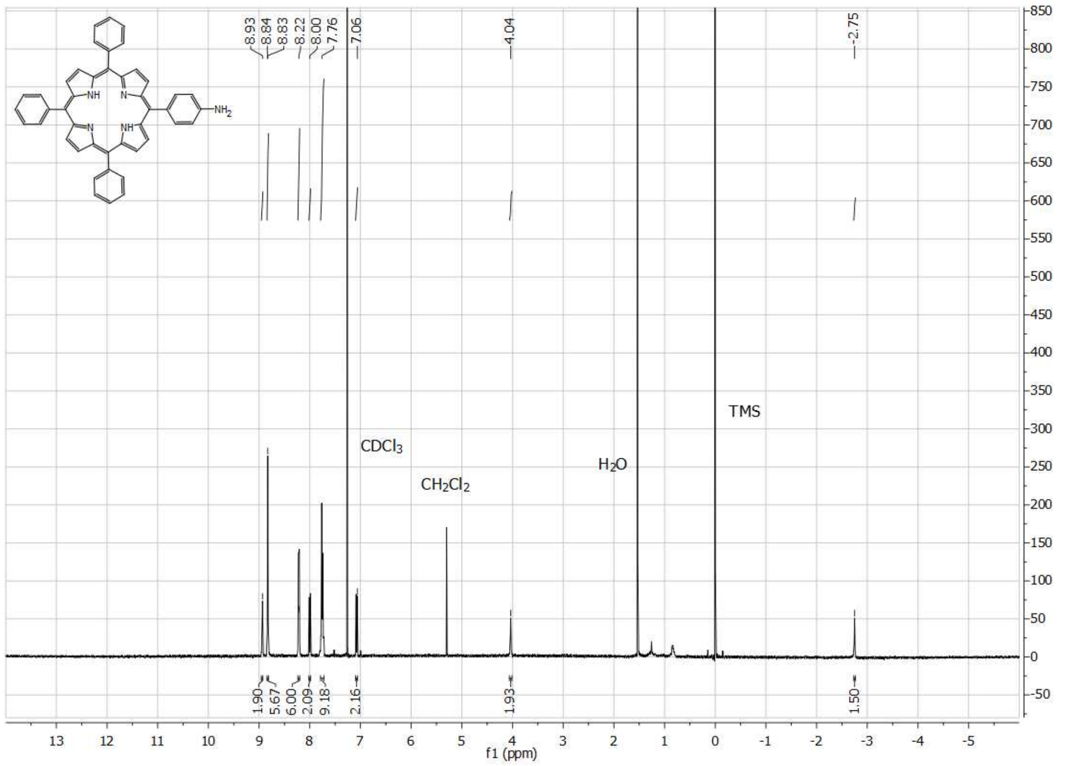

*5-(4-Aminophenyl)-10,15,20-tris(4-sulfonatophenyl)porphyrin trisodium salt,* ***2***

$^{1}$H NMR (400 MHz)

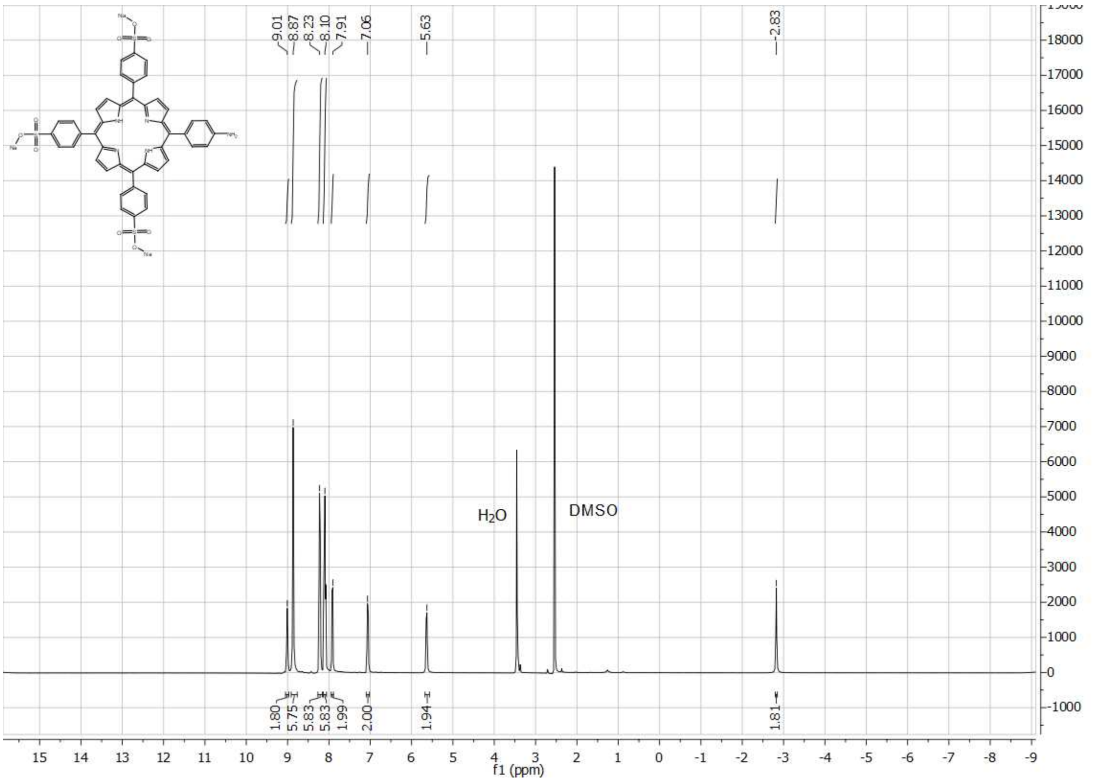

*5-Phenyl-10,15,20-tris(4-sulfonatophenyl)porphyrin trisodium salt,* **1**

$^{1}$H NMR (400 MHz)

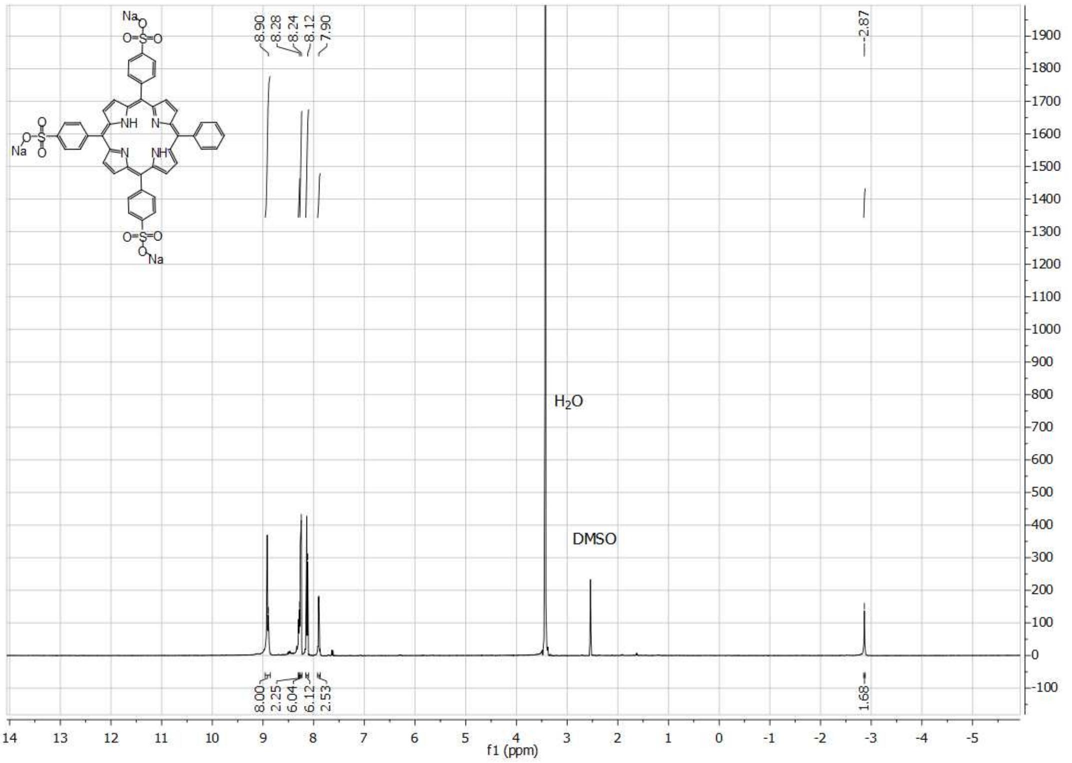


$^{13}$C NMR (100.6 MHz)

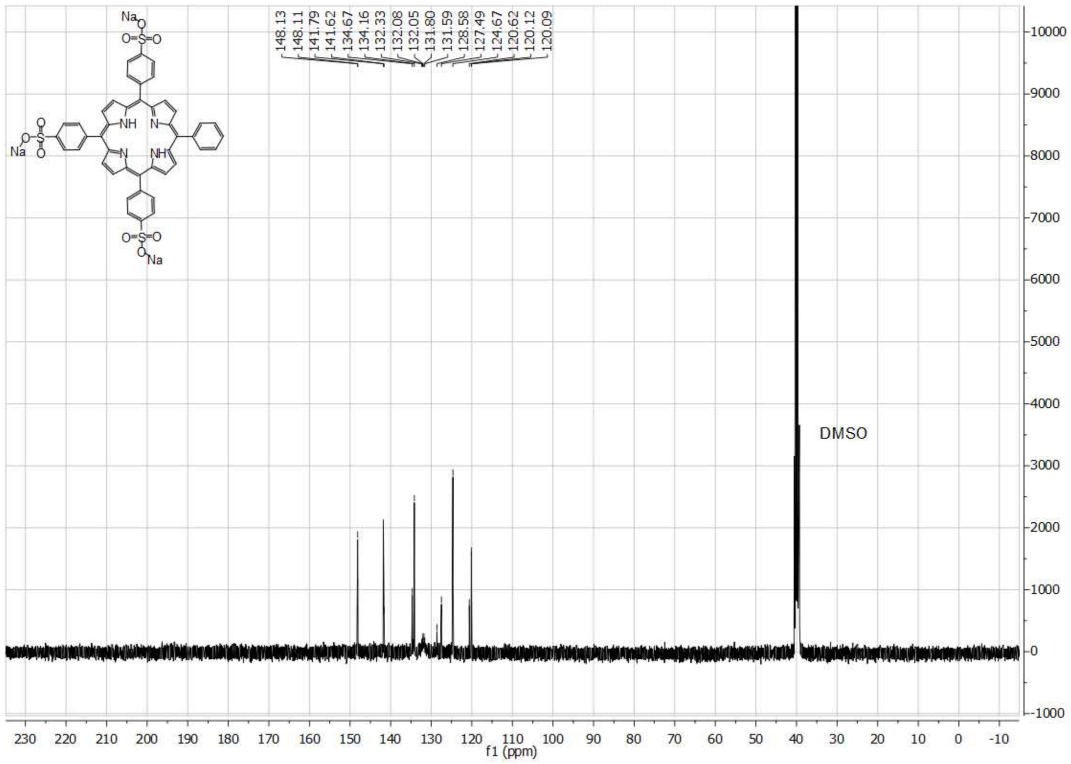

Diels-Alder adduct mixture **7a/b**

$^1$H NMR (400 MHz)

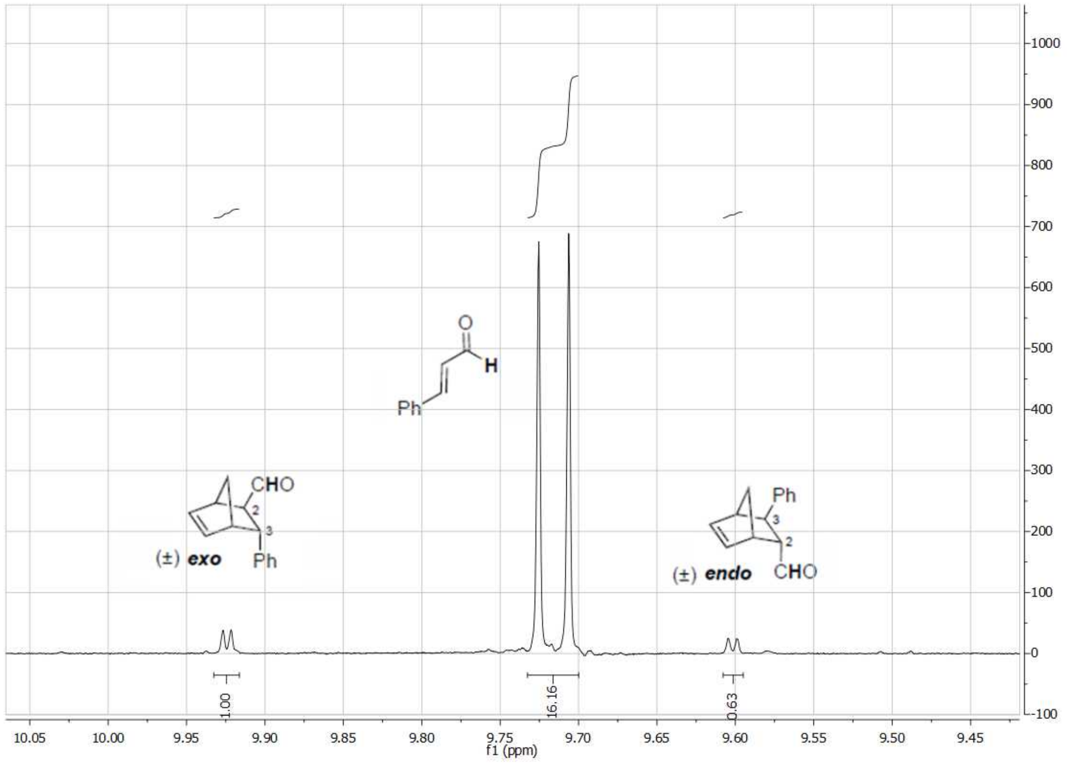

Supplement: Supplementary file 1 [file molecules-23-03363-s001.pdf]
